# Supplementary figures and images for: Impaired function and delayed regeneration of dendritic cells in COVID-19
Source: PLoS Pathog. 2021 Oct 6;17(10):e1009742. doi: 10.1371/journal.ppat.1009742 (PMC8523079; doi:10.1371/journal.ppat.1009742)

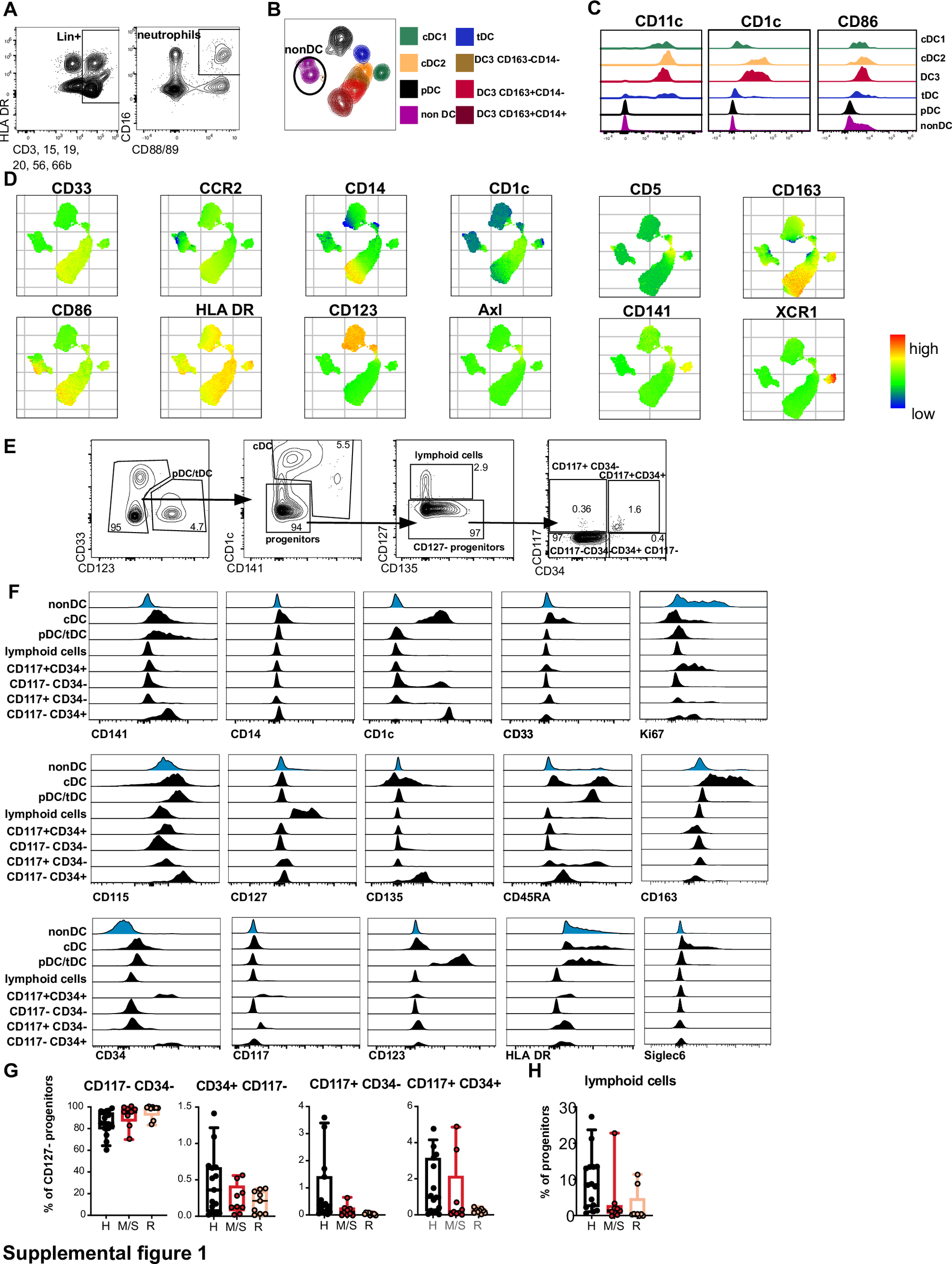

Supplement: S1 Fig — (A) Gating strategy for neutrophils in the blood: Within the lineage (CD3, CD15, CD19, CD20, CD56, CD66b) positive cells neutrophils were gated as CD16+ and CD88/89+. (B) UMAP clustering of one representative COVID-19 patient. Overlay of gated cDC1 (green, CD141+), cDC2 (orange, CD1c+, CD5+), DC3 (brown, red, dark red, CD1c+, CD5–, CD163+/–, CD14+/–), pDC (black, CD123+), tDC (blue, CD123+, Siglec1+, Axl+) and non-DC (purple, HLA-DR+, Lin-, CD141–, CD1c–) populations. (C) Representative histograms of CD11c, CD1c and CD86 expression in cDC1, cDC2, DC3, tDC, pDC and non-DC in a patient with moderate COVID-19. (D) Expression of several surface markers overlayed in the UMAP from (B). Shown is the expression of CD33, CCR2, CD14, CD1c, CD5, CD163, CD86, HLA-DR, CD123, Axl, CD141, XCR1 indicated by color scale (red = high expression, green = intermediate, blue = low expression). (E) Gating strategy for identification of progenitor populations in the blood. Cells are pregated on Lin−(CD3, CD15, CD19, CD20, CD56, CD66b, CD88, CD89), HLA-DR+ living cells. pDCs and tDCs are excluded via gating on CD123– cells followed by exclusion of cDCs by gating on CD1c–, CD141– cells. These progenitors are then separated into lymphoid cells (CD127+) and CD127– progenitors. Here, cells can be differentiated by their expression of CD117 and CD34 into four quadrants: CD117+ CD34–, CD117+ CD34+, CD117– CD34+, CD117– CD34–. (F) Representative histograms of marker expression of non-DCs, cDCs, pDC/tDCs, lymphoid cells, CD117+ CD34+, CD117– CD34–, CD117+ CD34–, CD117– CD34+ progenitor cells, gated according to (E) of one COVID-19 patient. Expression of CD141, CD14, CD1c, CD33, Ki67, CD115, CD127, CD135, CD45RA, CD163, CD34, CD117, CD123, HLA-DR and Siglec-6 is shown. (G) Percentage of CD117– CD34–, CD34+ CD117–, CD117+ CD34–, CD117+ CD34+ cells of total CD127– progenitor cells. (H) Percentage of CD127+ lymphoid cells of the progenitor cells are shown. Healthy donors (= H, black symbols, n = 12), m [file ppat.1009742.s001.tif]

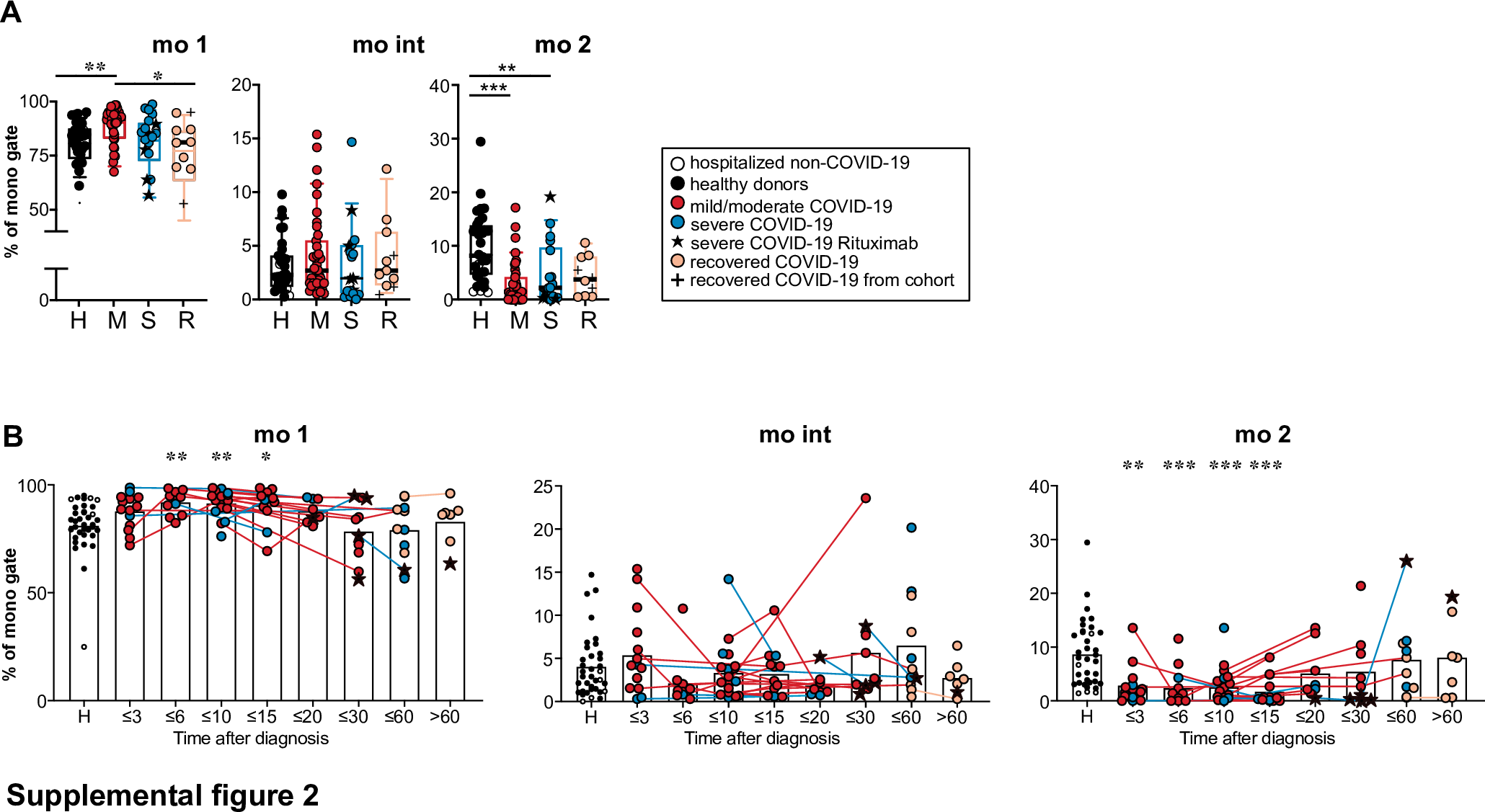

Supplement: S2 Fig — (A) Frequency of mo 1, mo int and mo 2 of all monocytes in healthy/non-COVID donors (H, n = 31), patients with mild/moderate (M, n = 39) and severe disease (S, n = 18) at the first analysis timepoint and recovered patients (R, n = 11). (B) Frequency of mo 1, mo int and mo 2 of all monocytes at different grouped timepoints after diagnosis. Connected lines represent multiple measurements of the same donor at different time points. Columns indicate the mean (Kruskal-Wallis test with Dunn’s correction, n = 124). * p<0.05, ** p> 0.01, *** p<0.001. (TIF) [file ppat.1009742.s002.tif]

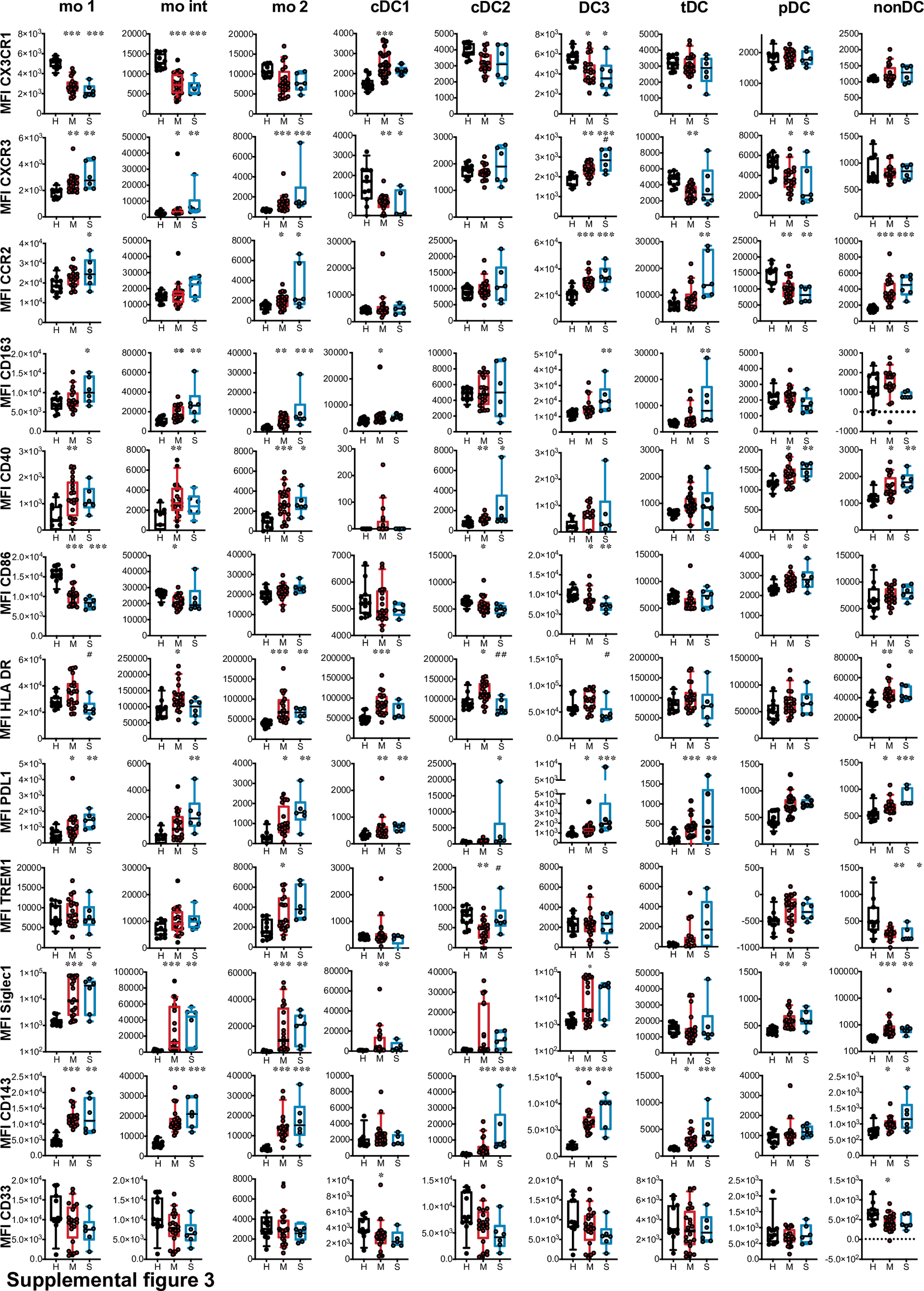

Supplement: S3 Fig — Expression of the indicated markers was measured as mean fluorescence intensity values in the indicated cell populations in COVID-19 patients with mild/moderate (M, n = 20) or severe disease (S, n = 6) at the first analysis timepoint compared to healthy donors (H, n = 11). Results for individual patients are indicated by symbols, Box plots show the 25 to 75 percentile; whiskers show the 10 to 90 percentile, horizontal lines indicate the median (Kruskal-Wallis test with Tukey’s or Dunn’s correction, n = * p<0.05, ** p<0.01, *** p<0.001). (TIF) [file ppat.1009742.s003.tif]

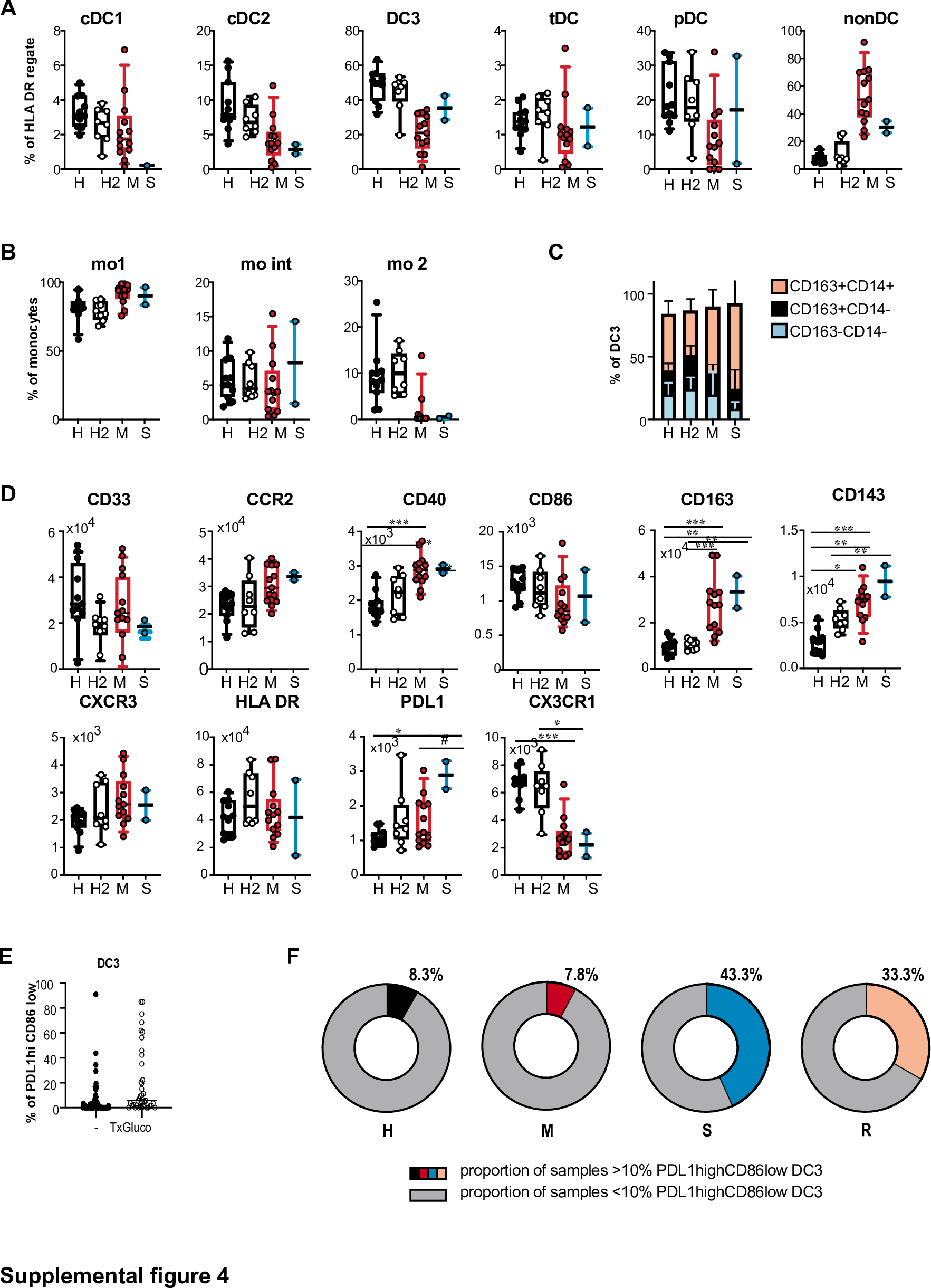

Supplement: S4 Fig — (A) Relative frequencies of DC subsets and non-DCs within the DC gate are shown. (B) Relative frequencies of classical monocytes (mo 1), intermediate monocytes (mo int) and non-classical monocytes (mo 2) within the monocyte gate are shown. (C) Relative frequencies of DC3 subtypes identified by CD163 and CD14 expression are shown (mean and SD). (D) Surface expression (MFI) of several markers shown in mo 1. (A-D) Healthy patients (= H1, black, n = 11), aged healthy patients (= H2, white, n = 6), mild/moderate COVID-19 pts (= M, red, n = 8) and severe COVID-19 patients (= S, blue, n = 2). Kruskal-Wallis Test with Dunn’s correction, or ANOVA and Tukey’s test was used, * p<0.05, ** p<0.01, *** p<0.001. (E) Frequencies of PD-L1hi CD86lo DC3 in patients receiving glucocorticoid therapy (white) and not receiving glucocorticoid therapy (black) are shown (n = 86). (F) Proportions of samples with more than 10% PD-L1hi CD86lo DC3 are shown as colored segments in the pie charts for patients with mild/moderate disease (M), severe disease (S), recovered patients (R) and controls (H, healthy donor and hospitalized non-CoV controls). (TIF) [file ppat.1009742.s004.tif]

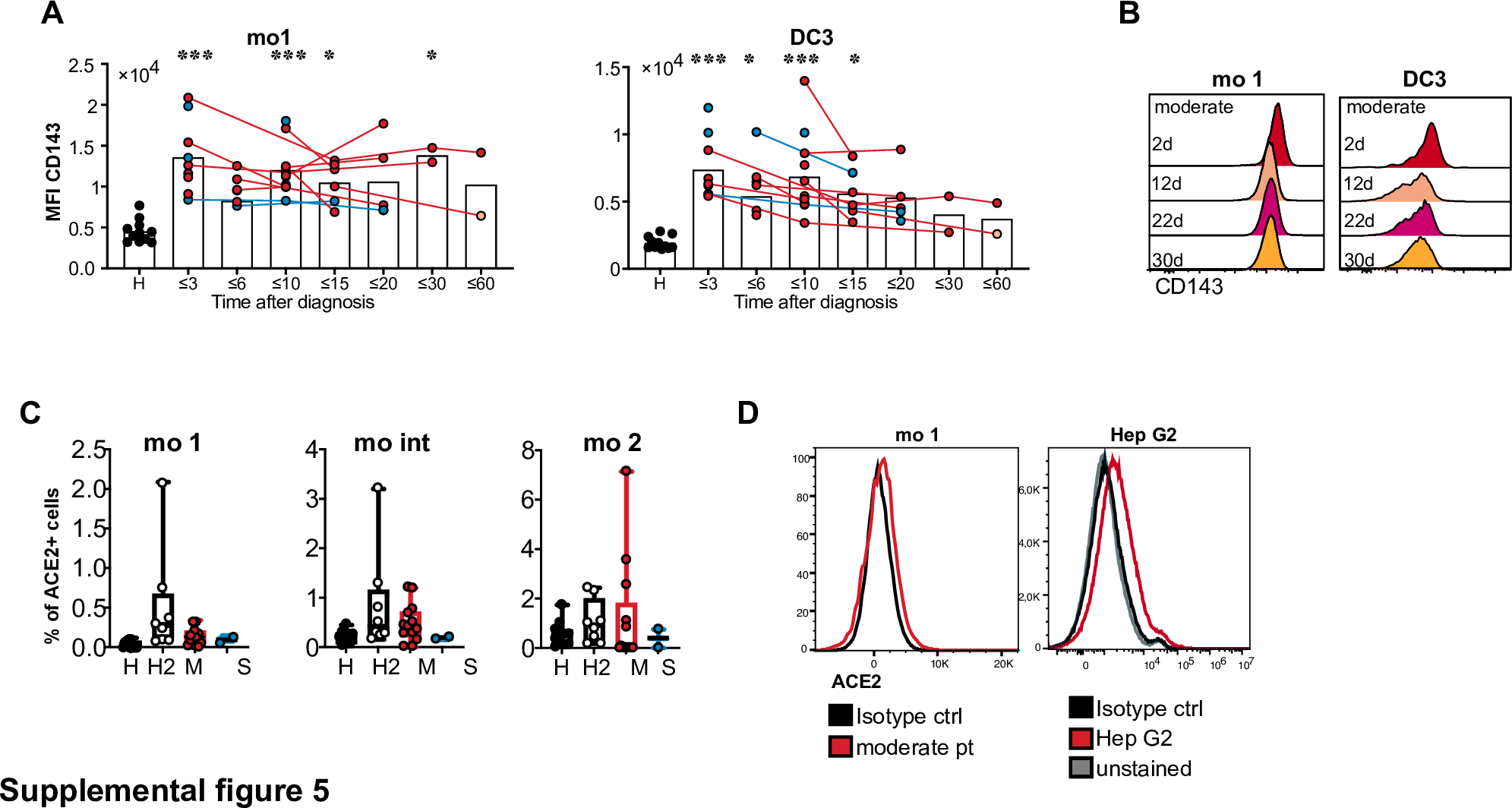

Supplement: S5 Fig — (A) CD143 expression (MFI values) at different timepoints after diagnosis in mo 1 and DC3. Connected lines represent multiple measurements of the same donor at different time points. Columns indicate the mean. Kruskal-Wallis test with Dunn’s correction. * p<0.05, ** p<0.01, *** p<0.001. (B) Representative histograms of CD143 expression in mo 1 and DC3 in a patient with moderate COVID-19 at the indicated time points after diagnosis. (C) Relative frequencies of ACE2-positive cells in mo 1, mo int and mo 2. (A-F) Healthy patients (= H, black, n = 11), aged healthy patients (= H2, white, n = 8), mild/moderate COVID-19 pts (= M, red, n = 13) and severe COVID-19 patients (= S, blue, n = 2). (D) Representative histogram of ACE2 expression in mo 1 in a moderate COVID-19 patient (red) and the isotype control (black) and of ACE2 expression in Hep G2 cells (red), unstained Hep G2 cells (grey) and the isotype control (black). (TIF) [file ppat.1009742.s005.tif]

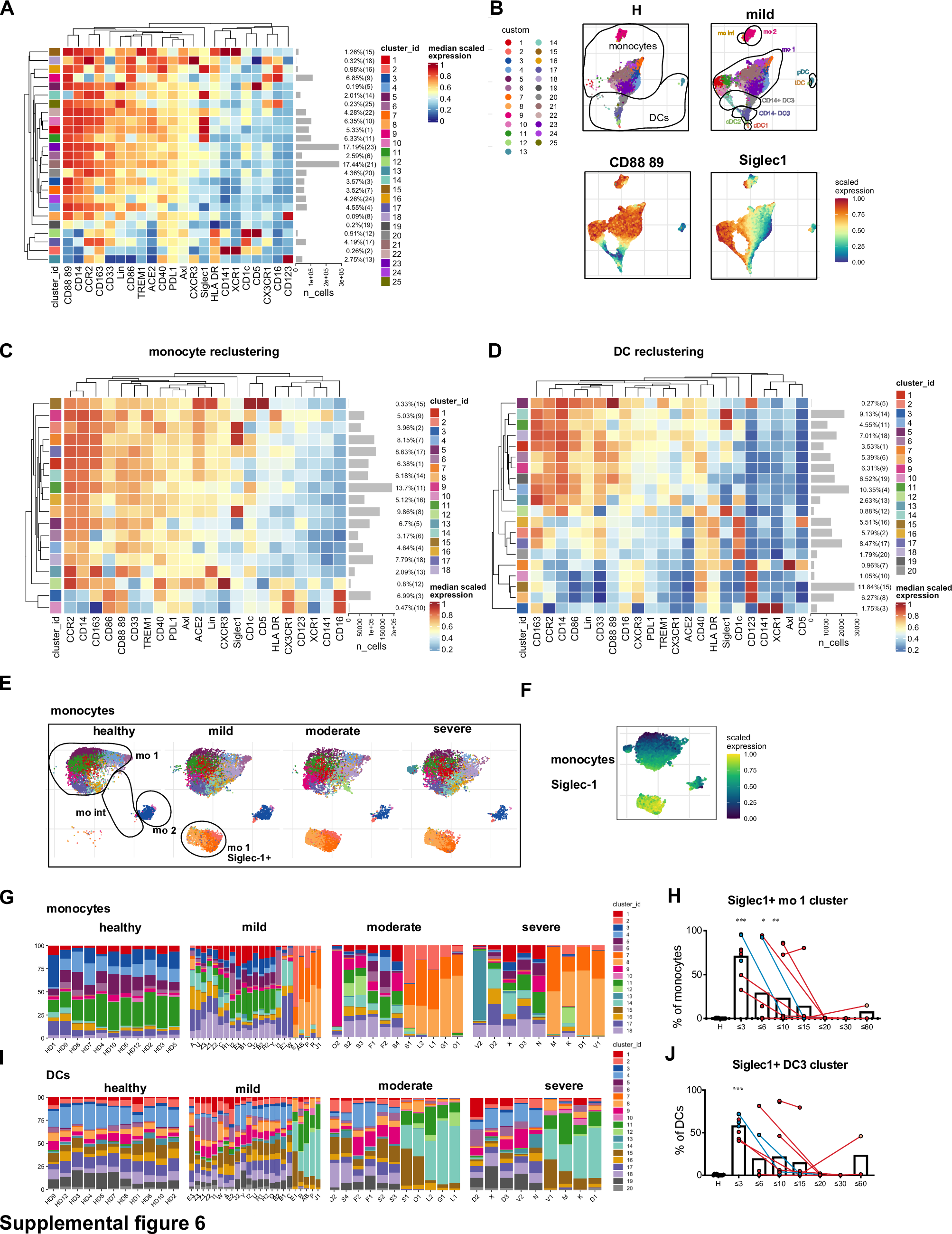

Supplement: S6 Fig — (A, B) Results of phenograph reclustering of HLA-DR+/intermediate Lin- cells (DCs and monocytes) after exclusion of all undefined cells. (A) Heatmap of marker expression in phenograph clusters and (B) UMAP of pooled data from healthy controls (H) and COVID-19 patients with mild disease are shown with phenograph clusters indicated by colors and annotation of monocyte and DC subpopulations indicated. Color overlays show the scaled marker expression for CD88/CD89 and Siglec-1. (C) Heatmap of marker expression in phenograph clusters of reclustered monocytes and (D) of reclustered DCs. (E) Monocyte UMAPs with phenograph clusters indicated by colors are shown separately for the indicated patient groups with annotation of monocyte subpopulations indicated in the UMAP of healthy controls. (F) Monocyte UMAP with color overlay indicating scaled Siglec-1 expression in pooled data from COVID-19 patients with moderate disease. (G and I) Frequencies of phenograph clusters derived from reclustering of monocytes (G) and DCs (I) in individual patients grouped by disease severity and controls. Letters indicate patients, numbers indicate consecutive sampling timepoints. Healthy donors (HD) were numbered. (H, J) Frequency of monocytes in the Siglec-1+ mo 1 cluster (H) and of DCs in the Siglec-1+ DC3 cluster (J) at the indicated grouped time points. Red symbols: mild/moderate COVID-19; blue symbols: severe COVID-19; orange symbols: recovered. Connected lines represent multiple measurements of the same donor at different time points. Columns indicate the mean. Kruskal-Wallis test with Dunn’s correction. * p<0.05, ** p<0.01, *** p<0.001. (TIF) [file ppat.1009742.s006.tif]

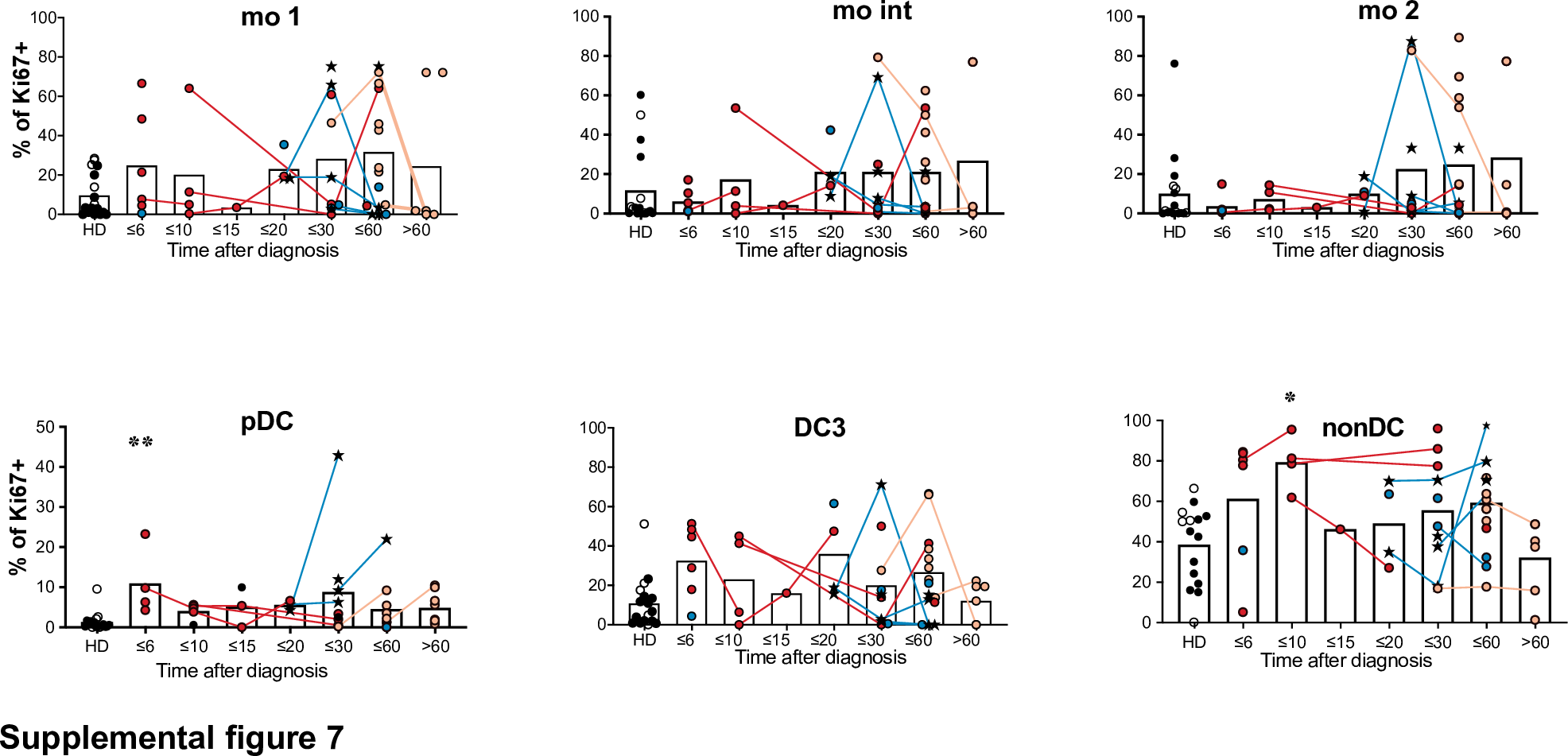

Supplement: S7 Fig — Frequencies of Ki67+ cells mo 1, mo int, mo 2, pDCs, DC3 and non-DCs at the indicated grouped time points. Results for individual patients are indicated by symbols as in Fig 2. Black: healthy donors; white: hospitalized non-CoV controls; red: mild/moderate COVID-19; blue: severe COVID-19; star: severe with B cell depleting therapy; orange: recovered. Connected lines represent multiple measurements of the same donor at different time points. Columns indicate the mean. Kruskal-Wallis test with Dunn’s correction. * p<0.05, ** p<0.01. (TIF) [file ppat.1009742.s007.tif]

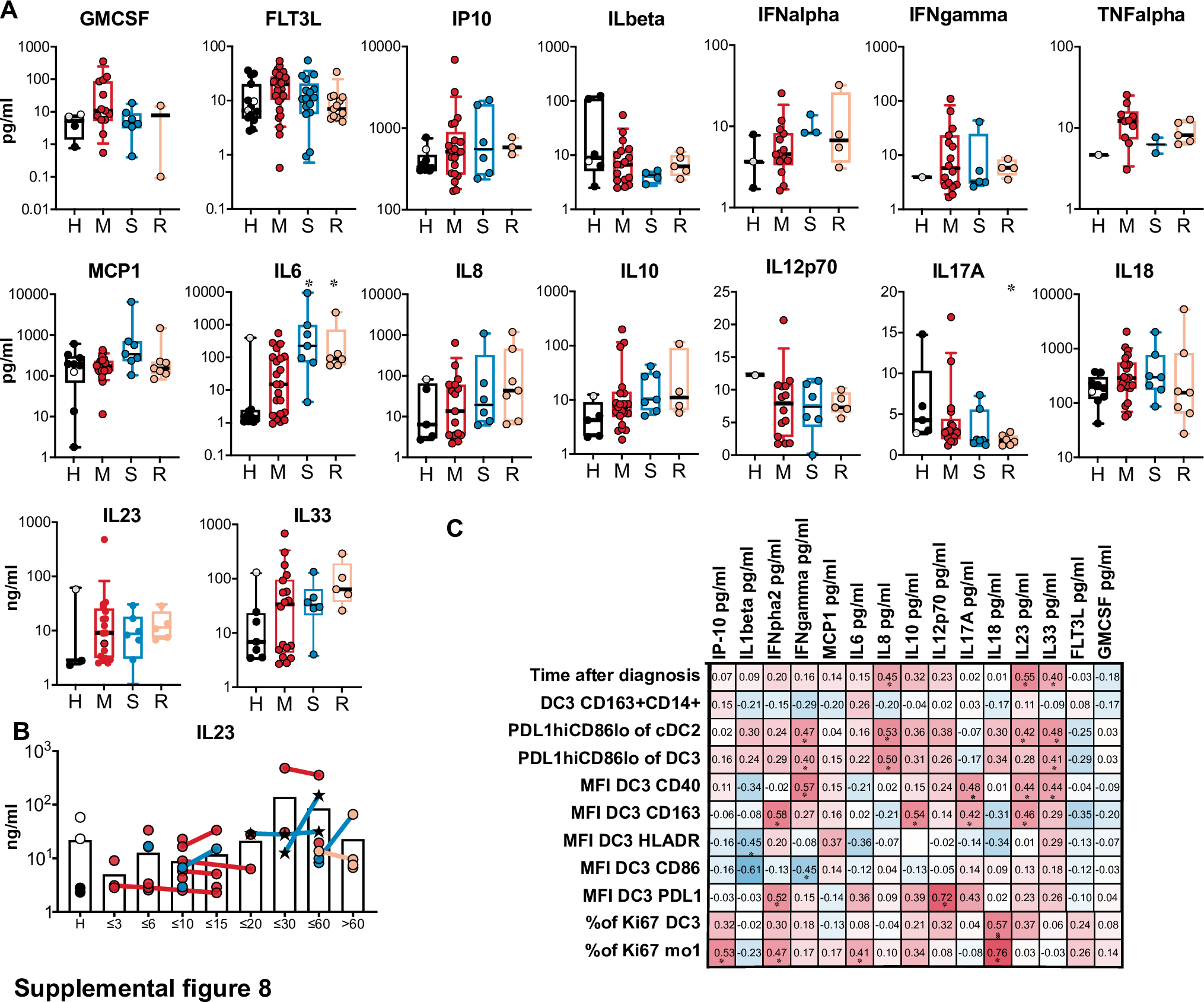

Supplement: S8 Fig — (A) Plasma concentrations (pg/ml) of plasma cytokines in healthy patients (= H, black, n = 1–15), mild/moderate COVID-19 pts (= M, red, n = 10–30), severe COVID-19 pts (= S, blue, n = 2–17) and recovered (= R, orange, n = 5–13) measured at the first timepoint after diagnosis. (B) Plasma concentrations (pg/ml) of IL-23 at different grouped time points after diagnosis. Connected lines represent multiple measurements of the same donor at different time points. Columns indicate the mean. Kruskal-Wallis test with Dunn’s correction, n = 44. * p<0.05, ** p<0.01, *** p<0.001. (C) Spearman correlation of plasma concentrations of cytokines at all timepoints with time after diagnosis, DC phenotype and Ki67 expression at the same sampling time points (n = 9–94, * p< 0.05). (TIF) [file ppat.1009742.s008.tif]

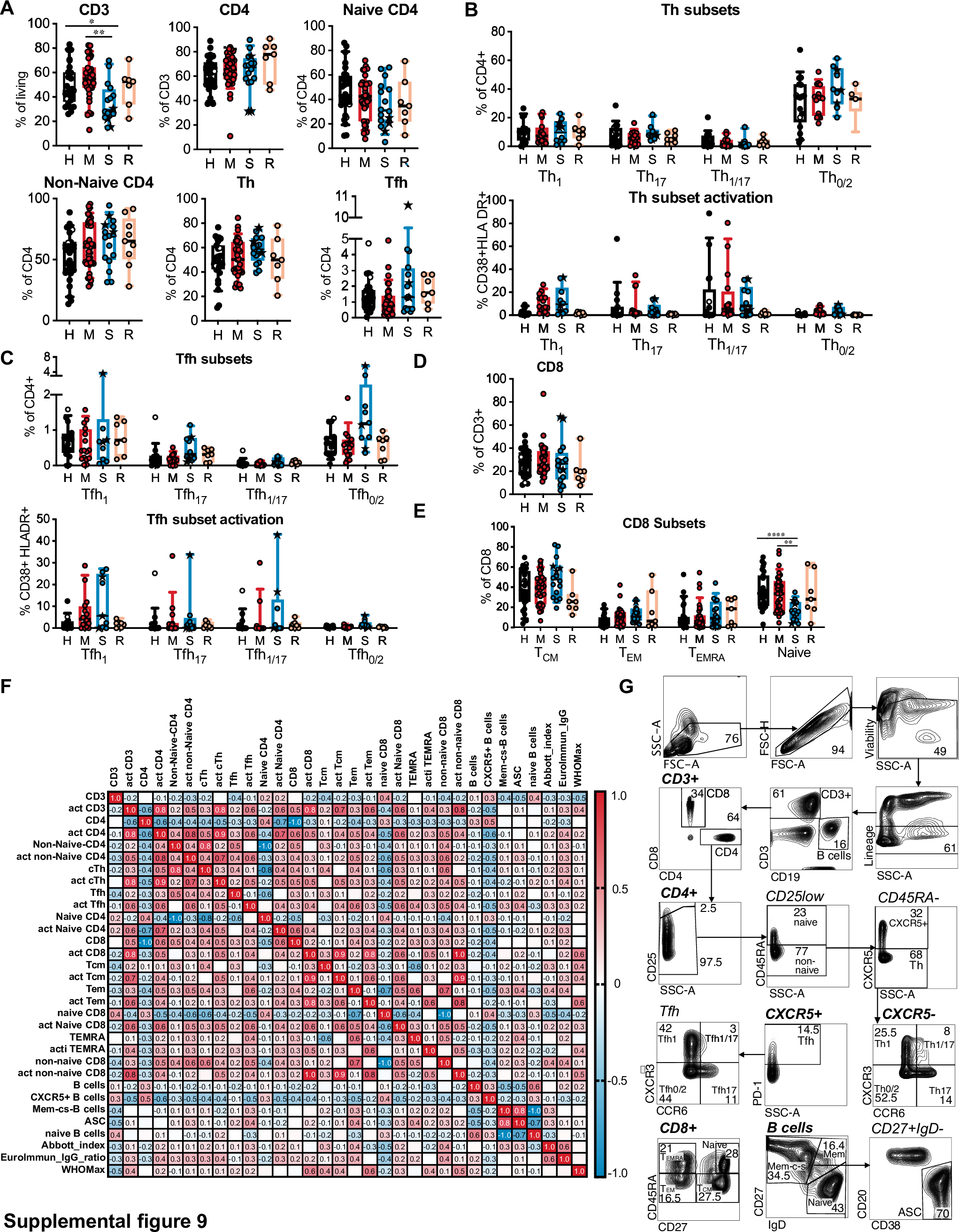

Supplement: S9 Fig — (A) Upper panel: Frequencies of the indicated T cell populations in healthy/non-COVID controls (H, n = 24) and acute COVID-19 patients with mild/moderate (M, n = 35) or severe disease (S, n = 16) at the first analysis timepoint and recovered patients (R, n = 7). Lower panel: Percentage of CD38+ HLADR+ activated cells within the indicated T cell subsets. (B) Upper panel: Frequencies of Th cell subsets (Th1: CXCR3+ CCR6–, Th17: CXCR3– CCR6+, Th1/17: CXCR3+ CCR6+, Th0/2: CXCR3– CCR6–) in CD4+ T cells. Lower panel: Percentage of CD38+ HLA-DR+ activated cells within the indicated Th cell subsets. (C). Upper panel: Frequencies of Tfh cell subsets in CD4+ T cells. Lower panel: Percentage of CD38+ HLA-DR+ activated cells within the indicated Tfh-like cell subsets. (B, C) H, n = 22; M, n = 16; S, n = 10; R, n = 7. (D) Percentage of CD8+ T cells. (E) Frequencies of CD8+ naïve and memory subsets within CD8+ T cells. (D, E) H, n = 24; M, n = 35; S, n = 16; R, n = 7. Kruskal-Wallis test with Dunn’s correction, * p<0.05, ** p<0.01, *** p<0.001. (F) Spearman correlation of adaptive parameters at time after diagnosis 10 to 25 days (n = 35–42). (G) Exemplary gating strategy for T and B cell subpopulations shown for one COVID-19 patient. (TIF) [file ppat.1009742.s009.tif]
