# Supplementary material for: Impaired function and delayed regeneration of dendritic cells in COVID-19
Source: PLoS Pathog. 2021 Oct 6;17(10):e1009742. doi: 10.1371/journal.ppat.1009742 (PMC8523079; doi:10.1371/journal.ppat.1009742)
Supplement: Attachment — Submitted filename: Winheim et al point by point reply.docx [file ppat.1009742.s015.docx]

**Point-by-point reply to the reviewers’ comments**

We would like to thank the reviewers for their time and effort in reviewing our manuscript and their valuable comments. We address each point raised by the reviewers below.

**Part II – Major Issues**

**Reviewer #3:**

*1. Many claims are being made without clearly indicating statistical significance or showing specific time-point. Actual p-values need to be shown so the readers can see if there is indeed a trend regardless whether or not such 'trend' has p-values less than or above 0.05.*

Reply: The actual p values are now provided as supplemental data in Table S3 and are given in the results text when there is a trend.

*In Fig2D, the authors claim that the increase in 'non-DCs' was "long lasting and observed even in recovered patients more than 60 days after primary diagnosis (page 5, line17-18)", but no statistical significance is shown between recovered donors more than 60 days after diagnosis* *and healthy donors.*

Reply: We found a significant increase in the percentage of non-DCs up to 20 days after diagnosis. As pointed out by the reviewer there was no significant difference in the percentage of non-DCs between patients sampled after 60 days post diagnosis and the control group, but a high frequency of non-DCs was observed in some patients also at later timepoints (Fig. 2 d). When comparing the percentages of non-DCs between the four groups shown in Fig. 2 c we found a significant difference between the recovered patients and the healthy donor controls (excluding the hospitalized non-CoV controls, p=0.0174). The results text was changed to describe these results more precisely.

*In Fig2E, "the frequencies of cDC1 and pDCs were significantly reduced at early time points (≤3 days after diagnosis) and largely restored in recovered patients (page 6, line 2-4)", but it shows that cDC1s are still significantly reduced at the time point over 60 days.*

Reply: As pointed out by the reviewer we found that in the patients that we sampled at more than 60 days after diagnosis the percentages of cDC1 (but not pDC) were also significantly reduced compared to controls. This is now stated precisely in the results text of the revised manuscript.

*In Fig4C, "the PD-L1/CD86 ratio in DC3 was increased in patients until late time points (page 7, line 21-22)" but there is only one time-point (≤10 days after diagnosis) that shows statistically significant difference compared to the healthy donor control.*

Reply: As pointed out by the reviewer a statistically significant difference in the PDL1/CD86 ratio (Fig. 4C) was only observed ≤10 days after diagnosis. “until late time points” was therefore omitted from this sentence. However, after excluding the hospitalized non-COVID-19 patients from the control group, a significant increase of CD86^lo^ PD-L1^hi^ DC3 and cDC2 (Fig. 4D) was also found in the group of recovered COVID-19 patients compared to healthy donors (DC3: p=0.0173, cDC2: p=0.0163) indicating that this phenotypic change was detectable beyond the acute phase of the disease. We point this out in the results text of the revised manuscript.

*In Fig4A, "CD33 was found to be downregulated in all APC populations of the patients especially in severe disease (page 8, line 23-24)", but the downregulation is mostly non-significant except for cDC1 in moderate diseases. Heatmap presentation is difficult to interpret since it does not visualize variances of the data.*

Reply: We agree with the reviewer that it is important to show the variance of the data in addition to the summary shown in the heatmap in Fig. 4 a. We therefore included graphs showing the individual data points and box plots for the MFI values of all markers in all monocyte and DC subsets in Supplemental Figure S3. Expression of CD33 was highly variable between the study subjects. A significantly reduced expression of CD33 was detected in cDC1 of patients with mild/moderate disease compared to healthy controls (Fig. 4 a, S3). In a subgroup of patients very low expression levels of CD33 were detected in the other DC subsets and in monocytes subsets, but there was no statistically significant difference between patients and controls. This is now described clearly in the results text of the revised manuscript.

*In Fig5A-D, "Increased Ki67 expression was detected in DCs and monocytes of recovered patients and even later than 60 days after diagnosis in some patients (page 11, line 8-9)", but there is no data showing any time-point in Fig 5A-D.*

Reply: Thank you for pointing this out. The kinetic of Ki67 expression is now shown in Supplemental Figure S7. We still detected a high percentage of Ki67^+^ mo 1, mo int, mo 2 and cDC1 in some recovered patients compared to controls (not statistically significant) suggesting enhanced cellular turnover beyond the acute phase of the disease. We performed the statistical analysis also after excluding the hospitalized non-COVID-19 patients, which showed higher proliferation, from the control group. We found a statistically significant increase in Ki67+ cells in DC3 and tDCs also in the recovered group compared to healthy donors. This is described in the results text of the revised manuscript and shown in Table S3.

*In FigS4, "the plasma concentrations of FLT3L and GM-CSF, growth factors, which can promote the generation and expansion of DCs and monocytes, were slightly higher in patients compared to heathly controls, especially in those with mild or moderate disease severity (page 11, line 11-14), but it is unclear if the increase is statistically significant and/or correlates with the Ki67 expression in any of the DC/monocyte subsets.*

Reply: As shown in Supplemental Figure S8 we detected no significant differences in Flt3L and GMCSF levels between the groups, only a trend towards higher Flt3L concentrations in the mild/moderate patient group (p=0.16). This is stated clearly in the text now and the p-value is given. Correlation analysis of the percentage of Ki67^+^ mo 1 and DC3 with cytokine concentrations was performed and the results were added to the correlation matrix shown in Supplemental Figure S8. We found no correlation with Flt3L and GM-CSF plasma concentrations, but with IL-18 plasma concentrations. These results are described in the results text of the revised manuscript.

*In Fig6D, “We also detected lower concentrations of the cytokines IL-2, IL-4, IL-5, IL-9, IL-10, IL-13, IL-17A, IFN-g, and TNF-a in cocultures of CD4+ T Cells and DC3 (page 13, line 4-6)” but the Figure only show T Cell + mo1 coculture rather than DC3 and only IL-10 shows statistically significant reduction in patient-derived samples.*

Reply: Thank you for pointing out this discrepancy, which is due to an error in figure labeling. The data shown in Figure 6 d is from cocultures with DC3 and not monocytes. We corrected this mistake and describe the results of statistical analysis in the results text of the revised manuscript.

*In FigS5A, "We observed a shift from naïve (CD45RA+) to non-naïve (CD45RA–) CD4+ T cells in the severe group (page 13, line 23-24), but it is unclear if the "shift" is statistically significant. Maybe the ratio between non-naive/naive would be more informative.*

Reply: We took up this suggestion and calculated the non-naïve/naïve CD4^+^ T cell ratio which also did not show a statistically significant difference. The results text was changed to state this correctly. In CD8^+^ T cells however a significant reduction in naïve T cells was observed in patients with severe disease vs controls and mild/moderate patients (as shown in Figure S9e). The ratio of non-naïve to naïve CD8^+^ T cells was also significantly increased in the mild/moderate and the severe groups vs the control group (see below).

**Ratio of non-naïve to naïve CD4+ and CD8+ T cells in patients with active COVID-19**

M: mild/moderate, S: severe, R: recovered patients and H: controls. H, n=24; M, n=35; S, n=16; R, n=7. Kruskal-Wallis test with Dunn’s correction, *p<0.05, **p<0.01, ***p<0.001.

*In FigS5B, "Th0/2 cell fraction was increased in patients with severe disease (page 14, line 8-9), but the data do not seem to indicate any statistically significant increase. Is this assessment based on something else such as correlation analysis?*

Reply: The difference was not statistically significant. We adapted the results section to state that only a trend towards increased Th0/Th2 cells was observed.

*In Fig8C, "We detected decreased naïve and memory but increased class-switched memory B cells compared to controls in a subgroup of COVID-19 patients", but again there seems to be no statistically significant changes shown in the graph. The authors need to explain why those "subgroup of COVID-19 patients" are defined in order to make this claim, since there is also a fraction of healthy donors who had low % (~20%) naive B cells and/or high % (>40%) class-switched memory B cells according to Fig8C.*

Reply: The difference was not statistically significant due to the high variance of the data. We could not detect a correlation with age or other clinical and biographical parameters that might have explained the variance. We adapted the results section accordingly to avoid overstating these results. The increase in IgD^–^ CD27^+^ CD38^+^ CD20^lo^ antibody secreting cells (ASC) was clear and statistically significant as shown in Figure 8d and e.

2. Some of the subset definitions are ambiguous or not shown in the data. cDC1, cDC2 and pDC *are described as a Lin- HLADR+ CD14- CD88/89- CD16- population (page 5, line4), but Fig1A does not show the CD14 negativity as a part of DC criteria (and CD14+ cells are classified as DC3 in Fig3).*

Reply: Thank you for pointing out this mistake, which we corrected in the results text. DC were gated as shown in Figure 2A as Lin- HLADR+ CD88/89- CD16-. CD14 negativity in pDCs and tDCs was confirmed (Figure S1) but not used as a criterium for defining pDCs or tDCs.

*The 'non-DC' is described as HLADR+CD86+/- (page 5, line 15) but the CD86 expression is not shown in Figures.*

Reply: CD86 expression in non-DC compared to other DC subsets is now shown in the Supplemental Figure S1 c. It is also shown as color overlay in Supplemental Figure S1 d.

*DC3, which is a part of DC, is defined as CD88/89 negative in Fig1A, but the cluster 11 in FigS3A-C, which is a part of CD14+CD163+Siglec1+DC3 (page 10, line 8) seems to be heavily positive for CD88/89.*

Reply: Cluster 11 was classified as monocytes not as DC3 (Supplemental Figure S6 a, b). Clusters 14 and 20 were annotated as CD14^+^ DC3, which express CD14, but only very low levels of CD88/89 compared to monocytes (see Supplemental Figure S6 a, b). This is now more clearly explained in the legend to Supplemental Figure S6 and we added the sentence “Phenograph clusters were assigned to the monocyte or DC compartment according to differential expression of CD88/89 (Fig. S6 b)“ to the results text.

*cDC2 and DC3 are gated on both CD1c+ and CD1c- cells in Fig1A, but their distinction criteria from the 'non-DC' based on the CD141 vs CD1c plot is unclear. Does the CD1c- cDC2/DC3 correspond to the CD141-CD11c- DC4 reported in Villani et al (Science 2017), which was later suggested to be CD16+ monocytes by Duterte et al (Immunity 2019), or are they instead related to non-DCs?*

Reply: CD1c expression was clearly negative in non-DC and positive in cDC2 and DC3 as shown in Figure 2a and Supplemental Figure S1c and d. cDC2 showed the strongest CD1c signal (see Supplemental Figure S1c) and DC3 show a continuum of low to high expression of CD1c (shown in Figure 2 a and Supplemental Figure S1c). This was also observed in the unbiased clustering analysis, where CD1c expression was highest in cDC2, lower in CD14^–^ DC3 and even lower in CD14^+^ DC3 (Supplemental Figure S6 a and b). The markers defining the subsets are now described in more detail in the results text to Figure 2.

*3. In the Abstract and Discussion, the authors suggest the contribution of changes in DC numbers and phenotype to the "immunosuppressed state" (page 17, line 13) of COVID19 patients. While such state may exist, it is not defined in their cohorts, and the data in fact suggests (hyper)activation of T cells (Fig7) and B cells (Fig8) rather than immunosuppression. I understand that hyperactivation of T/B cells and some forms of immunosuppression can potentially coexist, but correlation analyses between the changes in DC subsets and parameters for immunosuppression (such as lymphopenia, higher immunoregulatory cytokines, lower immunostimulatory cytokines, exhaustion markers on T cells) need to be added to Fig8H in order to make that claim.*

Reply: In the discussion we wrote: ”Thus, reduced APC numbers and functionality may contribute to an immunosuppressed state in COVID-19 patients, making them vulnerable to other infections or virus reactivation.“ We do not claim that the adaptive immune response to acute SARS-CoV-2 infection is suppressed. Based on our phenotypic and functional data we propose that DCs and monocytes could be impaired in inducing T cell responses to other pathogens, which may be encountered by COVID-19 patients during their illness or prolonged recovery period. To avoid misunderstanding of this sentence we changed it to: ”Thus, reduced APC numbers and functionality may impair *de novo* T cell activation in COVID-19 patients, making them vulnerable to other infections or virus reactivation.“

We show in Figure 4 i (correlation matrix) that PD-L1/CD86 ratio and PD-L1 expression especially in DC3 correlate with lymphopenia and inflammation (CRP, IL-6). Correlation of the DC3 phenotype with other plasma cytokines are shown in Supplemental Figure S8. PD-L1 expression correlated positively with IFN-alpha and IL-12p70, CD86 expression correlated inversely with IL-1β and IFN-γ. The frequency of PDL1^hi^ CD86^lo^ cDC2 and DC3 correlated positively with immunostimulatory IFN-γ, IL-8, IL-23 and with IL-33. No significant correlations of this DC3 phenotype with IL-10 were observed. We conclude that the PDL1^high^ CD86^lo^ phenotype is associated with elevated immunostimulatory cytokines and CRP in the plasma indicating systemic inflammation, but also with lymphopenia, which may be due in part to hyperinflammation.

As suggested by the reviewer we have performed correlation analyses with expression of co-inhibitory receptor PD-1 in T cells. It is controversial whether PD-1 expression indicates an activated, terminally differentiated or exhausted T cell state during the response to SARS-COV-2 infection {Rha, 2021 #231}. We found a correlation of PD-1 expression in T cells with PD-L1 expression in monocytes and to a lesser extent in DC3 in our patient cohort indicating that PD-1 – PD-L1 interactions could occur in COVID-19. However, it is not clear that T cells expressing PD-1 and other co-inhibitory receptors are really exhausted in COVID-19. In fact, SARS-CoV-2-specific T cells expressing PD-1 were shown to be activated and fully capable of responding to antigenic restimulation ^1, 2^. The results of these correlation analyses were included in Figure 7 i and are discussed in the revised manuscript.

**Part III – Minor Issues**

**Reviewer #3:**

*1. Many panels in Supplementary Figures are not properly explained or discussed in the main text. References to Supplementary Figures in the main text need to specify which panel(s) is being discussed.*

Reply: Thank you for pointing this out. We now refer to the subpanels of the supplementary figures in the revised manuscript and show the supplemental data in the order of their mention in the results text. Data, that was not described in the results text was deemed non-essential and omitted (former Supplemental Figure S2 a, b, c)-

*2. Several uncommon abbreviations are used without explanation. For instance, I would assume tDC means "transitional DC", which was first defined by Leylek et al (Cell Rep 2019). If that is the case, the paper needs to be cited. Similarly, "cs-mem B cells" (page 15, line 21) should be explained.*

Reply: Abbreviations are now fully explained when they are first mentioned and the reference for transitional DC (tDC) (Leylek et al.) was inserted when the term is first mentioned in the revised manuscript. The markers defining B cell subsets were inserted into the results text. The abbreviation mem-cs was avoided in the results text and is explained in the legend to figure 8.

*3. In FigS3, "The Siglec-1+ DC3 and mo 1 populations appeared only in samples from COVID-19 patients taken until 14 days after diagnosis and accounted for more than 50 % of the monocytes and DCs in 89% of the samples taken within the first 3 days after diagnosis" (page 10, line 11-14), but I am not sure which data this sentence is referring to, as no data in FigS3 shows specific time-points of the samples. In the legend, "reclusterednd", "reclusteredAPs" and "reclusteredith" need to be explained.*

Reply: We now show the percentage of cells in the Siglec-1^+^ DC3 within all DCs and Siglec-1^+^ mo1 cluster within all monocytes obtained from clustering analysis at the different grouped time points after diagnosis in Supplemental Figure S6 h and j and describe it in the results text of the revised manuscript. The legend to this supplemental figure was corrected.

*4. In page 9 (line22), Siglec-1 is described as "type 1 IFN-induced", but FigS4D shows no clear evidence for significant increase in IFN-alpha or IP10. Is there any evidence that Siglec-1 is indeed induced by type 1 IFN in this cohort?*

Reply: IFN-alpha2 was not significantly increased in the plasma in the subcohort of patients, where it was measured (Supplemental Figure S8). IFN-alpha was not measured in the cohort of patients, in which we analysed Siglec-1 expression. However, we obtained recently generated plasma protein data from this patient cohort where a panel of analytes was quantified in plasma samples using Proximity Entension (Assay Olink). Unfortunately, IFN-alpha itself was not included in the panel. Therefore, we calculated an IFN1 score from the abundance of 12 plasma proteins which included 11 proteins induced by type I IFN in human MoDCs and additionally IFNL1. Siglec-1 was analyzed separately. We found a strong correlation of the IFN1 score with the Siglec-1 surface expression (shown for DC3 in Figure. 4 h). Siglec-1 plasma protein abundance also correlated strongly with the IFN1 score. These results were included in figure 4 and Table S4.

*5. In page 8 (line 5-8), the following statement requires more context and/or statistical clarification: “Considering all samples measured, a sizable population of CD86lo PD-L1hi DC3 (> 10%) was found in 7.8 % of samples of the mild/moderate group, 43.3. % of the severe group, 33.3% of the recovered group and 8.3 % of controls (data not shown)” Is there statistically significant difference between healthy donor and patient groups?*

Reply: Thank you for pointing this out. A statistically significant difference was observed between severe and control and between recovered and control (Fisher’s exact test). (M vs Ctrl: n.s., S vs Ctrl: p=0.0013, R vs Ctrl: p=0.0390, Fisher’s exact test two-sided). We included the statistical analysis of these percentages in the revised results text and show the data in Supplemental Figure S4 f.

**Reviewer #4:**

*It was a bit irritating to jump to Figure S4 in the first description of the results. The authors might consider splitting the supplementary Figure 4 into 2 figures (one with the monocyte data, the others with the cytokine analysis from plasma.*

Reply: Thank you for the suggestion. We split this figure to separate the monocyte data from the cytokine data.

*When describing cell populations, the authors should include the definition of cells e.g. the FACS marker needed for characterizing a Tfh cell, TCM, Teff, T EMRA cell (e.g. page 14).*

Reply: Marker expression of the indicated cell types have been included in the results text of the revised manuscript.

*In addition, I was wondering why the authors describe plasma cells as antibody secreting cells (ASCs)? The authors should give a reason for that (and also should give a FACS marker set for the characterization of that cell type).*

Reply: Marker expression of the B cell subsets have been included in the results text of the revised manuscript. Antibody secreting cells (ASC) were defined as CD19^+^ IgD^–^ CD27^+^ CD38^+^ CD20^lo^ and encompass mainly plasma blasts and a smaller population of CD138^+^ plasma cells ^3, 4^. CD138 staining did not give reliable results from frozen samples and therefore we could not measure the frequency of CD138^+^ cells in all samples. The definition is now clearly stated in the results section of the revised manuscript.

*The authors should recheck the manuscript for the usage of the plural of dendritic cells: DC or DCs.*

Reply: This has been checked and corrected.

*The authors might extend the materials and methods section. The describe in the text that they used T cells from COVID-19 patients and cocultured them with DCs from healthy individuals. The M+M section does not state that the authors performed MLR settings.*

Reply: Both the T cells and the DCs and monocytes were isolated from the same individuals (healthy donors or COVID-19 patients) and used for the autologous cocultures. This is now written more clearly in the revised manuscript (results and methods section).

*Page 1, second paragraph:
Each DC subpopulations has specific….might read…. Each DC subpopulation has specific…

Page 2, first paragraph
DC3 in human blood share characteristics of both cDC2 and monocytes but are distinct in…might add commas … DC3 in human blood share characteristics of both, cDC2 and monocytes, but are distinct in

Page 10, second paragraph
Increased myelopoiesis has been described in COVID-19 patients (28, 35).To… empty space missing between the sentences

Page 11, first paragraph
DCs and the HLADR+ non-DC population had the highest frequencies of Ki67+ cells even in healthy/non-CoV controls which…please add comma before ‘which’:….DCs and the HLADR+ non-DC population had the highest frequencies of Ki67+ cells even in healthy/non-CoV controls, which…

Page 12, first paragraph
…were found for IFN-g, IL-8, IL-23 and IL-33 (Fig. S4)…. Please add comma after IL-23… were found for IFN-g, IL-8, IL-23, and IL-33 (Fig. S4).

Page 14, first paragraph
CXCR5+ PD-1+ Tfh-like cells within CD4+ T …the plus in CD4+ T cells should be up

..increased HLADR and CD38 expression (36) we investigated the coexpression of these molecules…. Please add comma after (36)… increased HLADR and CD38 expression (36), we investigated the coexpression of these molecules…

Page 15, second paragraph
…of CCR2, CXCR3, HLADR and CD40 in DC3 and monocytes correlated with…please add comma after HLADR…. of CCR2, CXCR3, HLADR, and CD40 in DC3 and monocytes correlated with…

Page 17, first paragraph
In this study, we provide an in depth characterization of DC and monocytes subpopulations in the blood of hospitalized patients with mild, moderate or severe COVID-19 compared with healthy controls….please change (i-depth and monocytes to monocyte)… In this study, we provide an in-depth characterization of DC and monocyte subpopulations in the blood of hospitalized patients with mild, moderate or severe COVID-19 compared with healthy controls.

blood in COVID-19 patients while… please add comma… blood in COVID-19 patients, while

lineage or Lineage?

Page 17, second paragraph
in the blood which occurred in…comma missing… in the blood, which occurred in

The reduction in DCs, which has also been observed in previous studies (16, 18, 19, 37) may be due to increased emigration from the blood and sequestration in tissues, auch as the inflamed lung or lymphoid tissues. ….please check for a comma before ‘may’ and the word auch -->such?... The reduction in DCs, which has also been observed in previous studies (16, 18, 19, 37), may be due to increased emigration from the blood and sequestration in tissues, such as the inflamed lung or lymphoid tissues.*

Reply: Thank you for reading our manuscript carefully and pointing out these errors which we have corrected in the revised manuscript.

References

1.	Rha MS*, et al.* PD-1-Expressing SARS-CoV-2-Specific CD8(+) T Cells Are Not Exhausted, but Functional in Patients with COVID-19. *Immunity* **54**, 44-52 e43 (2021).

2.	Shahbaz S*, et al.* The Quality of SARS-CoV-2-Specific T Cell Functions Differs in Patients with Mild/Moderate versus Severe Disease, and T Cells Expressing Coinhibitory Receptors Are Highly Activated. *J Immunol* **207**, 1099-1111 (2021).

3.	Garimalla S*, et al.* Differential transcriptome and development of human peripheral plasma cell subsets. *JCI Insight* **4**, (2019).

4.	Huber JE*, et al.* Dynamic changes in circulating T follicular helper cell composition predict neutralising antibody responses after yellow fever vaccination. *Clin Transl Immunology* **9**, e1129 (2020).
